# Pulmonary Atresia with Intact Ventricular Septum, a National Comparison Between Interventional and Surgical Approach, in Combination with a Systemic Literature Review

**DOI:** 10.1007/s00246-024-03566-x

**Published:** 2024-07-05

**Authors:** Stina Manhem, Michal Odermarsky, Håkan Wåhlander, Britt-Marie Ekman-Joelsson

**Affiliations:** 1 Department of Pediatrics, Institution for Clinical Sciences, Sahlgrenska Academy, University of Gothenburg, Gothenburg, Sweden; 2 Department of Pediatric Cardiology, Skåne University Hospital, Lund, Sweden; 3 Department of Clinical Sciences, Lund University, Lund, Sweden; 4 Department of Cardiology, Queen Silvia Children’s Hospital, Behandlingsvägen 7, 416 50 Gothenburg, Sweden

**Keywords:** Pulmonary atresia with intact ventricular septum, Valve lesions, Catheter-based valve perforation, Surgical valvulotomy

## Abstract

This study aimed to compare long-term morbidity in patients with pulmonary atresia with intact ventricular septum (PA-IVS) treated with catheter-based intervention (group A) versus those undergoing heart surgery (group B) as initial intervention. Additionally, we conducted a systematic literature review on PA-IVS treatment. All neonates born in Sweden with PA-IVS between 2007 and 2019 were screened for inclusion. The inclusion criterion was decompression of the right ventricle for initial intervention. Medical records were reviewed, as well as the initial preoperative angiogram, and the diagnostic echocardiogram. Comparisons between groups were performed with Mann–Whitney *U*-test and Fisher´s exact test. A systematic literature review of original studies regarding treatment of PA-IVS (2002 and onward) was conducted following the Preferred Reporting Items for Systematic Reviews and Meta-Analyses guidelines, to assess the outcomes of patients with PA-IVS. 34 (11 females) patients were included, 18 in group A and 16 in group B. There was no mortality in either group. Follow-up time ranged from 2 to 15 years (median 9). All attempted perforations in group A were successful, and 16 out of 18 patients reached biventricular circulation. In the surgical group 15 out of 16 patients reached biventricular circulation. The literature review presented heterogeneity in standards for treatment. This retrospective population-based multicenter study demonstrates that both catheter-based intervention and heart surgery are safe procedures. Our results are comparable to, or exceed, those in the systematic literature review. The systematic literature review displays a great heterogeneity in study design, with no definitive golden standard treatment.

## Introduction

Pulmonary atresia with intact ventricular septum (PA-IVS) is a rare and heterogeneous congenital heart defect. The morphology ranges from a hypoplastic right ventricle with a thick muscular atresia of the pulmonary valve, which renders the circulation to be univentricular with only a left ventricle, to a well-developed right ventricle with a thin membranous atresia capable of sustaining a biventricular circulation. In the latter type, initial decompression of the right ventricle with biventricular circulation is most often the aim, regardless of the presence of ventriculo-coronary artery connections (VCACs), given that the coronary circulation is not right ventricle dependent (RVDCC) [1, 2]. Surgical valvulotomy and cardiopulmonary bypass has been associated with morbidity and mortality [3, 4]. Catheter-based approach instead of surgery in the first days of life is less invasive and has become the standard practice in many centers over the past three decades [5–9]. The pulmonary valve can be perforated through laser, radiofrequency (RF) treatment or through mechanical perforation. In Sweden, catheter-based treatment of the pulmonary valve in PA-IVS was introduced in 2007 and has since been routinely performed at one of the two pediatric cardiac centers in the country.

The aim of this retrospective study was to compare the long-term morbidity of patients treated with catheter-based therapy with those who underwent heart surgery as the initial intervention for right ventricular decompression. Additionally, we conducted a systematic literature review to assess the concurrence of our findings with current international studies.

## Materials and Methods

### Retrospective Population-Based Multicenter Study

#### Patient Population

This is a retrospective population-based multicenter study that screened all neonates born in Sweden with PA-IVS between 2007 and 2019 for inclusion. Patients were identified through the Swedish National Register of Congenital Deformities (which has a 100% national coverage for PA-IVS), registers at pediatric cardiac centers and SWEDCON—the Swedish Registry of Congenital Heart Disease. There are two pediatric cardiothoracic centers in Sweden, with one center, since 2007, primarily using catheter-based intervention for patients with favorable morphology, while the other center prefers heart surgery. The allocation of patients to each center is determined by a national agreement, where Sweden's 21 regions independently decide which center to refer patients to for pediatric cardiac surgery. The severity or morphology of the heart defect does not influence the treatment center assignment, and the catchment areas have equal population sizes. The decision of where a patient is treated is solely based on the place of birth, making this study pseudorandomized.

#### Interventional Description

Catheterizations were performed under general anesthesia. Venous and arterial access was obtained from femoral vessels. Right heart and aortic pressures were documented prior to angiography. Right ventricular angiograms were performed with cranial angulation of the anterio-posterior plane to delineate the right ventricular outflow and to exclude right ventricular-dependent coronary circulation. Arterial duct and main pulmonary artery locations were determined on aortic angiography. A 5F Judkins 2.5 right coronary catheter was placed in the right ventricular outflow for optimal positioning of the RF-wire. A Nykanen RF perforation wire (Baylis Medical, Mississauga, ON) with a Baylis RF generator was used. Serial hand injections were used to approach the center of the pulmonary valve plate using both A–P and lateral projections. We have used a snare catheter placed in the main pulmonary artery via a retrograde approach through the ductus arteriosus during RF perforation to assist in aligning the RF catheter with the pulmonary valve. An RF pulse was applied at 7 Watts for 2 s to perform the perforation. Successful perforation was confirmed by capture of the perforation catheter with the snare or with injection of contrast. A 0.038-inch ProTrack microcatheter was advanced on the RF-wire, which then was exchanged for a 0.014-inch exchange wire. The exchange wire was secured in either the distal pulmonary tree or, most often, across the patent ductus arteriosus (PDA) into the descending aorta. In the aorta, the guidewire was often secured with the snare to enhance stability of the wire. Pulmonary valvuloplasty was then performed, often with a Tyshak Mini (NuMED Inc, Cornwall, ON) balloon. Balloon diameter was chosen to be 120–140% of measured valvular diameter. Pre-dilation was only preformed in a few patients early during the experience. Post-dilation pressure waveforms were recorded simultaneously in the right ventricle and the aorta. A repeat right ventricular angiogram was performed prior to completion of the procedure. Prostaglandin E1 infusion was regularly stopped at the end of the procedure. Stenting of the arterial duct or surgical systemic-to-pulmonary shunt was reserved for patients that failed repeated attempts of weaning from prostaglandin E1 infusion and was consequently performed as a later and separate procedure. The choice of this treatment strategy is motivated by the objective to minimize radiation exposure, reduce the dose of contrast agent, and achieve optimal hemodynamic stability.

#### Surgery

Surgical valvotomy was performed and additional source of pulmonary blood flow in form of modified Blalock-Taussig shunt was added when deemed necessary. Concomitant atrial septectomy was performed in selected cases.

#### Reintervention

A reintervention has been defined as a subsequent intervention (catheter-based or surgery) performed after a successful opening of the pulmonary valve.

#### Methods

All patients underwent an initial echocardiogram and those who were deemed unfit for biventricular repair, i.e., the right ventricle was not decompressed, were excluded from the study.

The inclusion criterion was decompression of the right ventricle for initial intervention. Patients were eligible for initial decompression if they had membranous pulmonary atresia and a bi- or tripartite right ventricle. VCACs were not an exclusion criterion, but RVDCC was. The presence of tricuspid valve dysplasia, of various degree, was noted but not an exclusion criterium. The initial preoperative angiogram, if performed, and the diagnostic echocardiogram were reviewed. Medical records were reviewed regarding weight, length, reinterventions, complications, time admitted to hospital and time in the pediatric intensive care unit (PICU) in connection with the initial intervention. The follow-up period for reinterventions and complications ended in March 2022.

The echocardiographic examinations were reviewed by pediatric cardiologist MO, pediatric resident SM, pediatric consultant BE and pediatric consultant HW. Spot-checks were conducted to minimize the risk for bias. Dr HW reviewed the angiograms performed at Queen Silvia Children's Hospital in Gothenburg, there were no angiograms available for review at Skåne University Hospital. The pulmonary regurgitation (PR) and tricuspid regurgitation (TR) were estimated by eyeballing on echocardiogram with color Doppler.

The two pediatric cardiothoracic centra have different primary treatment method. We compared the patients treated with catheter-based intervention and balloon dilation of the pulmonary valve to those who underwent heart surgery for initial intervention. The diagnostic echocardiograms were compared between the two groups along with outcomes related to final circulatory status, complications, number and type of reinterventions, and mortality.

#### Statistics

Continuous variables were analyzed using the Mann–Whitney *U*-test and are presented as mean and standard deviation. Categorical variables were analyzed using the Fisher’s exact test and are expressed as numbers and percentages. Statistical significance was set at a *p*-value < 0.05. Statistics software R version 4.2.1 was used for the analyzes.

### Systematic Review of Current Literature

A systematic literature review was conducted following the PRISMA (Preferred Reporting Items for Systematic Reviews and Meta-Analyses) guidelines to assess the outcomes of patients with PA-IVS who underwent initial intervention for right ventricle decompression. The search was performed in November 2022 using the following keywords, separate and in combination: “pulmonary atresia”, “pulmonary valve atresia”, “pa ivs”, “paivs”, “pa-ivs”, “intact ventricular septum”. We searched the English and Swedish literature with Medline and Embase databases in a combined search and the Cochrane database for all studies published between 2002 and 2022. Reviews were not included but their reference lists were examined to identify relevant original studies that met the inclusion criteria but were not found through the initial search. Inclusion criteria were original studies that focused on initial treatment of PA-IVS performed from 2002 and onwards. Studies published before year 2002 were excluded, to avoid results that were obsolete. Exclusion criteria were case reports, duplicates of previously published data and studies that included patients without prerequisites for biventricular circulation or where the morphology of the right ventricle was not clear. A few studies comprised patients with both severe hypoplastic right ventricles and well-developed right ventricles suitable for biventricular repair. If the subgroups were clearly separated and the data presented separately, the group of patients with biventricular morphology was included in the review. If the subgroups had not been analyzed separately the study was excluded. In a similar manner, studies that included both critical pulmonary stenosis (CPS) and PA-IVS were included only if the subgroups were analyzed separately, the group with PA-IVS was then included. This approach was chosen to ensure comparability of results, as the success rate of perforation may differ in studies that include patients with CPS.

## Results

### Retrospective Population-Based Multicenter Study

#### Patient Characteristics

Sixty seven patients with PA-IVS were born in Sweden during the study period and were reviewed for inclusion. For a comprehensive understanding of the assessment of prerequisites for biventricular circulation or univentricular palliation in the two different pediatric thoracic centers, see Tables 1 and 2. 34 patients (11 females) were included in the study. 18 patients (4 females) were initially treated with catheter-based treatment (group A) and 16 (7 females) were initially treated with surgical decompression of the right ventricle (group B). Three patients were intended for catheter-based treatment by mechanical perforation but had a failed valve perforation. In the analyses they were included in group B.

**Table 1:** Patient characteristics and echocardiogram measurements preoperative for patients whose right ventricle was not decompressed

Variable	Treatment hospital							*p*-value
	Queen Silvia Children's Hospital in Gothenburg (*N* = 12)				Skåne University Hospital, Lund (*N* = 21)			
	Count	Median		Valid *N*	Count	Median	Valid *N*	
Female gender	5 (42%)			12	10 (48%)		21	
Weight (kg)		3.26 (2.79, 3.44)		12		3.31 (2.89, 3.65)	21	0.42
Body surface area		0.21 (0.19, 0.22)		12		0.21 (0.19, 0.23)	19	0.63
Type of atresia								0.024*
Membranous	2 (17%)			12	12 (57%)		21	
Muscular	10 (83%)			12	9 (43%)		21	
VCACs	7 (70%)			10	9 (60%)		15	0.69
TV diameter		7.5 (5.8, 9.2)		12		6.7 (5.6, 9.6)	17	0.84
* Z*-score		− 2.08 (− 3.21, − 1.37)		12		− 2.43 (− 2.75, − 0.31)	14	0.58
TV diameter/ MV diameter ratio		0.50 (0.41, 0.68)		12		0.57 (0.47, 0.82)	16	0.32
PV diameter		5.00 (4.00, 6.00)		10		6.40 (5.75, 7.15)	15	0.017*
* Z*-score		− 2.33 (− 2.72, − 1.57)		10		− 1.52 (− 2.01, − 0.85)	14	0.035*
Main pulmonary artery		6.00 (5.50, 7.00)		10		7.20 (6.20, 8.07)	18	0.067
Circulation type								
Univentricular	11 (92%)			12	17 (81%)		21	0.63
1,5-Ventricular	0			12	1 (4.8%)		21	> 0.99
Biventricular	1 (8.3%)			12	3 (14%)		21	> 0.99

All valve and vessel diameters are specified in millimetres. Body Surface Area is measured in square meters, calculated using the Boston Children's Hospital *Z*-Score Calculator. Data are reported as count (percentage) or median (IQR). *p-value < 0.05

*VCACs* ventriculocoronary artery connections, *TV* tricuspid valve, *MV* mitral valve, *PV* pulmonary valve, *(1,5-ventricle)* open pulmonary valve and bidirectional Glenn anastomosis

**Table 2:** Patient characteristics and echocardiogram measurements preoperative for patients whose right ventricle was decompressed

Variable	Patients treated with catheter-based intervention (*N* = 18)			Patients treated with heart surgery (*N* = 16)			*p*-value
	Count	Median	Valid *N*	Count	Median	Valid *N*	
Female gender	4 (22%)		18	7 (44%)		16	
Weight		3.87 (3.40, 4.15)	18		3.30 (2.89, 3.58)		0.018*
Body surface area		0.24 (0.22, 0.25)	18		0.22 (0.19, 0.23)		0.038*
Type of atresia							
Membranous	18 (100%)			16 (100%)			
Muscular	0			0			
VCACs	0			3 (23%)			0.064
TV diameter		11.50 (10.25, 14.00)	18		12.00 (10.75, 13.00)	16	0.79
* Z*-score		− 0.04 (− 0.66, 1.28)	18		0.52 (− 0.50, 1.05)	16	0.66
TV diameter/ MV diameter ratio		0.93 (0.85, 1.09)	18		0.95 (0.78, 1.04)	16	0.67
PV diameter		6.95 (6.00, 7.00)	18		7.00 (6.88, 7.85)	16	0.11
* Z*-score		− 1.67 (− 2.02, − 1.39)	18		− 0.96 (− 1.10, − 0.47)	16	0.002*
Main pulmonary artery		9.45 (8.25, 10.00)	18		8.20 (7.45, 9.25)	16	0.021*
Dysplasia of TV	3 (17%)			2 (12%)			> 0.99
Circulation type							
Univentricular	0		18	1 (6.3%)		16	0.47
1,5-Ventricular	2 (11%)		18	0		16	0.49
Biventricular	16 (89%)		18	15 (94%)		16	> 0.99

All valve and vessel diameters are specified in millimetres. Body Surface Area is measured in square meters, calculated using the Boston Children's Hospital *Z*-Score Calculator. Data are reported as count (percentage) or median (IQR). *p-value < 0.05

*VCACs* ventriculocoronary artery connections, *TV* tricuspid valve, *MV* mitral valve, *PV* pulmonary valve, *(1,5-ventricle)* open pulmonary valve and bidirectional Glenn anastomosis

All included patients had a membranous atresia and a bi- or tripartite right ventricle. In group A and B the preoperative tricuspid valve (TV) *z*-score median was − 0.22 (− 0.66, 1.18) and 0.61 (− 0.03, 1.17) respectively. Preprocedural echocardiographic measurements are presented in Table 2. Three patients in group B had VCACs, no patient had RVDCC. Five patients had tricuspid valve dysplasia, three in group A and two in group B.

There were few associated extracardiac malformations or genetic disorders. Two patients had trisomy 21. One patient had undescended testicle, one patient had hip joint luxation, one patient had cortisol deficiency.

#### Interventional Technique

In group A the initial intervention consisted of a catheter-based valvotomy. Followed by a balloon dilation of the valve. No patient received additional source of pulmonary blood flow placed during the initial intervention.

In group B all patients underwent surgical valvulotomy. Additionally, during the initial intervention five patients underwent atrioseptostomy, one patient had a fenestrated patch placed in the ASD. Nine patients had a modified Blalock-Taussig shunt (mBTS), three patients had a transannular patch repair, two patients had a homograft to replace the pulmonary valve, one patient had a dilation of the pulmonary bifurcation, one patient had the PDA ligated.

#### Outcomes

There was no mortality in either group during the entire study period. One patient was lost to follow-up after discharge. Excluding this patient, follow-up time ranged from 2 to 15 years (median 9 years, 6, 13).

In group A, all attempted perforations with radiofrequency of the pulmonary valve were successful. Two out of five of the attempted mechanical perforations were successful. The 3 patients that had a failed mechanical perforation were included in, and their outcome was analyzed with, group B. 16 out of 18 patients reached biventricular circulation as the end result while 2 patients had a one-and-a half ventricle (1,5-ventrice) repair (open pulmonary valve and bidirectional Glenn anastomosis) at the end of the study. In group B, 15 out of 16 patients reached biventricular circulation while one patient required univentricular palliation.

In group A, 30 days after their initial intervention eight patients had no additional pulmonary blood flow and ten patients had additional pulmonary blood flow through either a mBTS or a PDA-stent, see Table 3. In group B 30 days after their initial intervention four patients had no additional pulmonary blood flow and twelve patients had additional pulmonary blood flow through a mBTS. Four patients with tricuspid valve dysplasia achieved biventricular circulation (3 in group A and one in group B), one patient in group B ended up with univentricular circulation.

**Table 3:** Reinterventions, outcomes, and complications 30 days after initial intervention

Variable	Patients treated with catheter-based intervention (*N* = 18)		Patients treated with heart surgery (*N* = 16)		*p*-value
	Count	Valid *N*	Count	Valid *N*	
Additional interventions performed during initial intervention	0	18	13 (81%)	16	< 0.001
Reintervention within 30 days after initial intervention	10 (56%)	18	4 (25%)	16	0.34
Outcome 30 days after initial intervention					
Valvulotomy alone	8 (44%)	18	3 (19%)	16	0.31
Valvulotomy + patch in the pulmonary valve annulus	0	18	1 (6.3%)	16	0.49
Valvulotomy + additional source of pulmonary blood flow^a^	10 (56%)	18	12 (75%)	16	0.78
Complication within 30 days after initial intervention	7 (39%)	18	3 (19%)	16	0.47
Of which occurred periprocedural	1 (5.6%)	18	0	16	1

^a^Additional source of pulmonary blood flow; Blalock-Taussig shunt, Patent Ductus Arteriosus-stent

Data are reported as count (percent)

One patient in group B was lost to follow-up after hospital discharge due to living abroad.

#### Length of Hospital Stay

Patients in group A had a tendency towards fewer days in the intensive care unit during their initial procedural admission, i.e., when they had their initial intervention, compared to patients in group B, median 3 (0, 11) and 7 (3, 17) days, respectively (*p*-value 0.11). Seven patients in group A were not admitted to the intensive care unit at all. The patients in group B exhibited a tendency towards a shorter overall hospital stay compared to group A, median 18 (10, 20) and 21 (15, 36) days, respectively (*p*-value 0.078).

#### Reinterventions

##### Group A

**Fig. 1:**
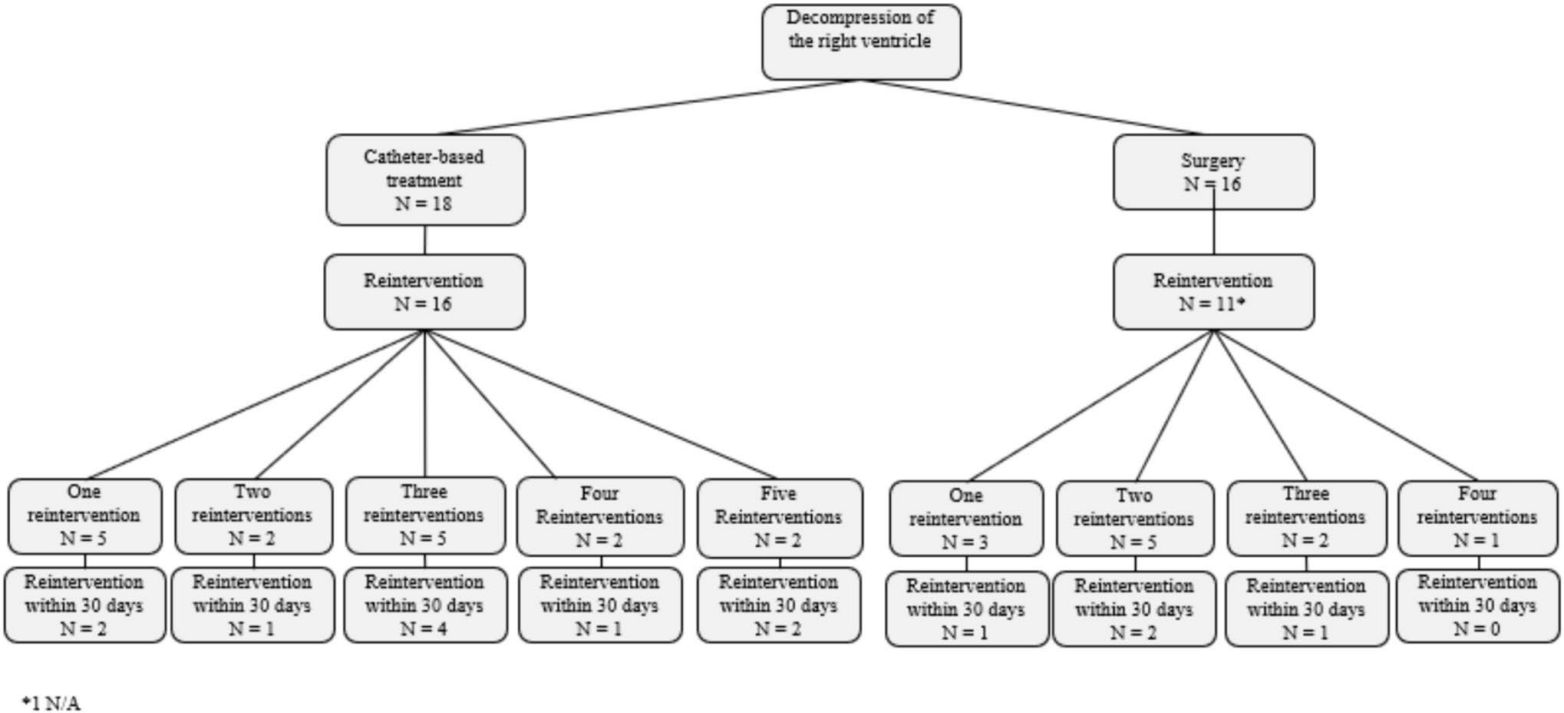
Overview of reinterventions

16 out of 18 patients in group A had at least one reintervention, see Fig. 1. Ten patients had a reintervention within 30 days after their initial intervention, five had a re-catheterization where they received a PDA-stent and five patients underwent heart surgery to facilitate additional pulmonary blood flow, see Table 3. 14 patients underwent at least one re-catheterization. Eleven patients underwent heart surgery after the initial catheterization. Nine patients had moderate-severe tricuspid regurgitation (TR) preoperatively, three of these patients needed additional pulmonary blood flow after initial catheterization, none of the patients that needed a reintervention within 30 days after initial intervention had tricuspid valve dysplasia. During long-term follow-up (after the initial procedural admission), there were in total 24 catheter-based reinterventions and 16 surgical.

##### Group B

Eleven out of 16 patients in group B had at least one reintervention, of whom four patients had a reintervention within 30 days after the initial intervention, all of which were surgical, see Table 3. Nine patients received a mBTS in their first intervention in addition to the surgical valvulotomy. Six patients had at least one catheter-based intervention. Ten patients had at least one reoperation. Six patients had moderate-severe TR preoperatively, one of whom required reoperation within 30 days after their first surgery, this patient had tricuspid valve dysplasia. During long-term follow-up, there were in total nine catheter-based reinterventions and 14 surgical.

#### Complications

In group A, one patient experienced a periprocedural complication, a right ventricle perforation and atrial flutter during catheterization, this resolved spontaneously, the patient was discharged after 14 days. Furthermore, within 30 days after their initial intervention, six patients experienced complications, including three shunt occlusions in arterial pulmonary shunts (AP-shunts) (one of which led to circulatory collapse and brain hemorrhage) one case of cerebral infarction, one case of necrotizing enterocolitis and acute kidney failure, and one case of infection. The patients who received a PDA-stent had no complications related to the stents.

In group B, three patients had a failed balloon valvulotomy before undergoing heart surgery. Within 30 days after the initial intervention, three patients in group B experienced complications: one patient had diaphragm paresis, one patient had anuria and needed dialysis, one patient had a stroke, suffered arrythmia, had a deep vein thrombosis, needed dialysis and ECMO and also suffered necrosis of fingers and toes.

### Systematic Review of Current Literature

**Fig. 2:**
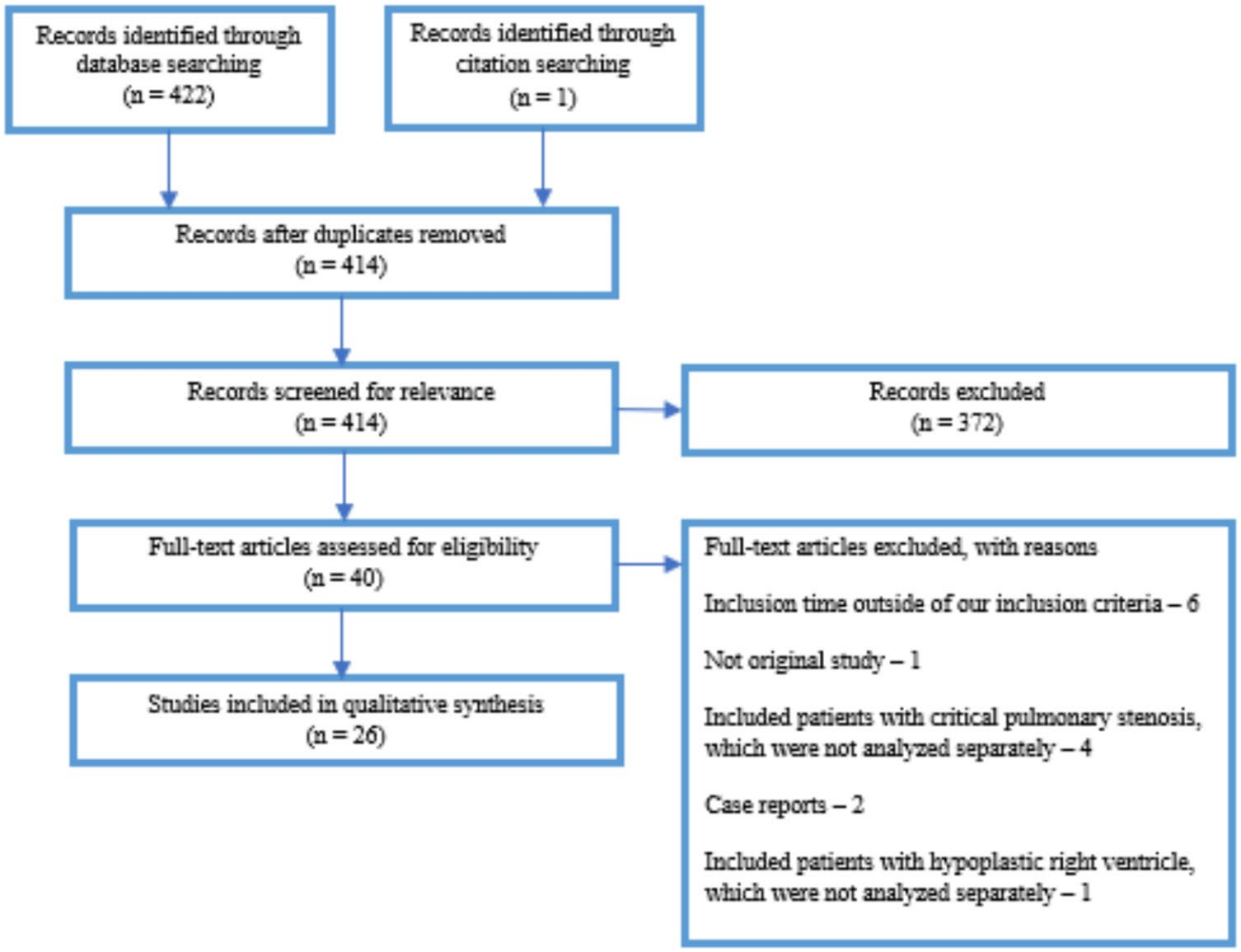
Systematic literature search according to Preferred Reporting Items for Systematic Reviews and Meta-Analyses (PRISMA)—guidelines

422 articles were identified, after duplicates were removed 414 articles remained and their abstracts were screened for relevance, see Fig. 2. All studies were assessed in regard to inclusion criteria and after that 25 studies were included in the review. Partially the same patient population were studied in two studies [10, 11]. In order to avoid analyzing duplicate results, data have been collected from all studies but each variable from each patient population was only analyzed once, see Table 4. The objectives, settings, inclusion criteria and *z*-score models of the included studies differed, and a meta-analysis could therefore not be performed.

**Table 4:** Summary of systematic literature review

Study	Number of participants	Primary way of treatment	Successful opening of the pulmonary valve	Reintervention post procedure	Complication peri- or post procedure	Early mortality^a^
Alwi (2013) [6]	8	Catheter^M^	7	2	1 Infection, not known what kind	0
Bakhru (2017) [12]	20	Catheter^M^	16	10	2 Perforation of the pericardium, of whom 1 tamponade	3
Brown (2017) [13]	5	Catheter^RF^	5	2	1 Perforation of the pericardium	0
Cho (2013) [2]	9	Catheter^M^	7	10	3 Artery thrombosis	0
El Saiedi (2018, 2022)^b^ [10, 11]	50	Catheter^M, RF^	39	9	7 Perforation of the pericardium	19
El Shedoudy (2018) [14]	13	Catheter^M^	11	1	1 Sepsis, 1 Heart failure, 2 Artery thrombosis	2
Haddad (2021) [15]	18	Catheter^M^	14	7	5 Perforation of the pericardium, of whom 4 tamponades	2
Hascoët (2019) [8]	29	Catheter^RF^	23	16	2 Perforation of the pericardium with tamponade, 1 Stroke, 4 Septic shock, 1 NEC, 1 AKI, 1 Hypothyroidism^c^	7
Hu (2015) [16]	22	Hybrid	19	2	1 Low cardiac output syndrome, 1 Ventricular tachycardia	2
Kamali (2021) [17]	26	Catheter^M, RF^	22	5	2 Vascular complications, 1 Cardiac arrest	3
Kim (2015) [18]	7	Catheter^M^	7	4	1 Atrial fibrillation	0
Lamers (2012) [9]	11	Catheter^RF^	11	3	4 Thrombosis	0
Lawley (2021) [19]	15	12 Catheter^U^ 3 Surgery	10 Catheter 3 Surgery	6	Catheter: 4 Hemopericardium, of whom 3 tamponades	2
Lefort (2019) [20]	5	Catheter^M^	5	2	0	0
Guanhua (2020) [21]	15	Surgery	15	NA	NA	0
Li (2013) [22]	10	Hybrid	10	1	1 Arrythmia, 2 Bleeding	0
Li (2011) [23]	30	Hybrid	30	1	0	0
Lin (2017) [24]	38	Hybrid	38	0	8 Pneumonia, 3 Right heart failure, 2 AKI, 3 Arrythmia	3
Odemis (2013) [7]	8	Catheter^RF^	8	NA	NA	NA
Patil (2016) [25]	9	Catheter^M^	8	1	1 Perforation of the pericardium with tamponade	1
Prakoso (2022) [26]	12	Catheter^M^	12	NA	1 Supra ventricular tachycardia, 1 Tamponade and cardiac arrest	0
Shaath (2012) [27]	20	Catheter^RF^	NA	11	NA	NA
Song (2022) [28]	100	Surgery/ Hybrid	100	0	2 Hypoxemia, 2 Right heart failure, 1 Pneumonia, 1 Sepsis	6
Yoldas (2020) [29]	31	Catheter^RF^	31	16	5 Arrythmia, 3 Arterial thrombosis, 2 Perforation of the pericardium	6
Zampi (2014) [30]	24	17 Surgery 7 Hybrid	24	2	Surgery: 3 Arrythmia, 2 Cardiac arrest, 1 AKI, 3 infections, 4 NEC, 1 other^d^ Hybrid: 4 Arrythmia, 2 Cardiac arrest, 3 AKI, 5 Infections, 5 NEC, 1 other^d^	0

Only first author is given for each study

*M* mechanical perforation of the pulmonary valve, *RF* radiofrequency perforation of the pulmonary valve, *U* unknown type of catheter used for perforation of the pulmonary valve, *NEC* Necrotizing enterocolitis, *AKI* Acute kidney injury

^a^Death during the first hospitalization, ^b^2 articles, ^c^Several smaller complications not specified further, ^d^Not defined further

### Patient Characteristics

The inclusion criterion in several studies was based on TV *z*-score, it ranged from > − 5 to > − 1 [9, 11, 12, 14, 17, 29]. All patients were considered to have a favorable morphology of the right ventricle, suitable for biventricular repair. A few patients had a muscular atresia or a hypoplastic right ventricle [11, 23]. tricuspid valve dysplasia was an exclusion criterion in several studies [14, 16].

### Intervention Approaches

The included studies evaluated different intervention approaches including surgical decompression of the right ventricle, hybrid interventions, radiofrequency perforation with subsequent balloon dilation of the pulmonary valve, and various mechanical perforation techniques using chronic total occlusion (CTO)-wires and coronary guidewires. Different studies have investigated the possibility to use the soft or the stiff end of different coronary wires and if a retrograde approach instead of the antegrade, which is standard in most centers, can have benefits [2, 6, 11, 12, 14, 15, 17, 18, 20, 25, 26]. The hybrid approach has emerged as an alternative to percutaneous treatment, aiming to reduce the perforation risk and enabling the placement of a mBTS if necessary without requiring additional interventions. The technique involves a midline sternotomy and under transesophageal echocardiographic guidance the pericardium and pulmonary valve is perforated with a needle and balloon pulmonary valvuloplasty is then performed [16, 22–24, 30]. Cardiopulmonary bypass (CPB) is avoided and the ductus arteriosus can be snared, if the patient then desaturates a stent in the ductus or a mBTS can be placed.

### Outcomes

#### Success Rate and Mortality

The success rate of catheter-based intervention in perforating the pulmonary valve ranged between 74 and 100% [2, 6–10, 12–15, 17, 18, 20, 25, 26, 29] and for hybrid approach 86–100% [16, 22–24, 28, 30, 31], see Table 4. Failed intervention was correlated to lower age, weight and BSA [10]. The early mortality (death within 30 days post procedure or death during the first hospitalization) varied from 0 to 44.1% across studies (hybrid procedure 0–9%, catheter-based procedure 0–44.1%, surgical procedure 0–12%) [10, 12, 14–17, 19, 21, 24, 25, 29]. The leading cause of death in several studies was infection or sepsis [11, 15, 24, 25, 29]. Prolonged stays in the PICU were found to be associated with higher mortality rates, even if the patients initially survived the intervention.

The length of follow-up varied among the included studies, ranging from no follow-up after discharge from hospital to 5.4 years [6, 7, 9, 11–25, 29, 30].

### Predictors and Likelihood for Biventricular Circulation or Reintervention

Several studies have focused on identifying predictors of biventricular circulation and the likelihood of reintervention or need for additional pulmonary blood flow [7, 9, 19, 22, 24, 27, 29]. Reintervention has been defined in various ways across the included studies, the most common type of early reintervention involves additional pulmonary blood flow, either through a mBTS or a PDA-stent [6, 9, 11–17, 19, 20, 22, 23, 25, 29]. Lawley et al. found that a small TR preoperative was negatively correlated with biventricular circulation [19]. Prakoso et al. deemed patients with a severe TR unfit for biventricular circulation [26].Shaath et al. showed that a peak gradient above 43 mm Hg of the TR was a risk factor for reintervention [27]. A larger tricuspid valve (TV) *z*-score was associated with greater right ventricle growth and served as a good predictor for biventricular circulation [29]. El Shedoudy et al. showed that there can be significant growth of the TV-diameter *z*-score after intervention [14]. Shaath et al. did, however, see no significant change of the tricuspid valve *z*-score, indicating that the right ventricular growth was not affected by the intervention [27]. A small pulmonary valve (PV) and TV showed to be a risk factor for reintervention [29]. Hu et al. state that a small PV annulus is not possible to enlarge through repeated balloon dilation, but the intervention will only result in a large pulmonary regurgitation (PR) [16]. El Saiedi et al. argue that a larger balloon/valve ratio and repeated balloon dilations with the aim to lower post-procedural right ventricle pressure (RVP) might benefit right ventricle growth and reduce the risk for reintervention [10]. The size of the pulmonary regurgitation has been suggested to be smaller after a hybrid procedure or surgical valvulotomy compared to after a catheter-based balloon dilation of the pulmonary valve [23, 28]. Authors to studies evaluating the hybrid approach mention that the balloon dilation can render a severe pulmonary regurgitation and they therefore argue to use a smaller maximal diameter of the balloon [16, 23, 24]. Song and Chen found that when perforating the pulmonary valve and balloon dilating the valve, in the setting of a hybrid intervention, the perforation needle can be deviated from the intended perforation point which could add to the risk for pulmonary regurgitation [28].

Patil et al. noticed that the oxygenation of the patients did not improve significantly until 2–3 days after the intervention [25]. Li et al. demonstrated that saturation does not normalize immediately after decompression due to residual outflow tract obstruction [22]. Postoperative hypoxemia could result from muscle edema in the right ventricle and the limited capacity of a hypoplastic right ventricle to support sufficient pulmonary blood flow [28]. Post-procedure RVP was directly proportionate to the need for prostaglandin [10]. In several centers, if patients cannot be weaned off prostaglandin after a set number of days and their saturation remains below 70–80%, the ductus arteriosus is stented or an mBTS is placed [9, 12, 15]. In other institutions a PDA-stent or a mBTS is placed in the same session if the saturation is unsatisfactory when the ductus is closed, or certain morphologic features are present [6, 12, 13]. If a PDA-stent is placed during the initial intervention there are fewer early reinterventions [11]. Li et al. showed that hybrid therapy carries a reduced risk of reintervention compared to catheter-based treatment [23]. In their study all infants aged under a month old had their ductus arteriosus ligated and a mBTS placed.

### Complications

The complication rate differed between the included studies in frequency and in severity, see Table 4.

### Cost

Radiofrequency perforation of the pulmonary valve is a more expensive intervention compared to mechanical perforation but is less costly than heart surgery. Mechanical perforation with various types of wires has been developed as a cost-efficient alternative, utilizing multipurpose equipment that is more readily available in cardiac centers with budgetary limitations [6, 11, 12, 14, 15, 17, 18, 22, 25].

## Discussion

### Retrospective Population-Based Multicenter Study

In our study we found that decompression of the right ventricle could be performed safe, with no associated mortality, using either catheter-based intervention or heart surgery.

It was a tendency towards shorter time in the PICU for the patients who were treated with catheter-based intervention initially (*p*-value 0.11). A reduced PICU stay can have a positive effect on the parent–child bonding parents [32]. Heart surgery may hinder practices like kangaroo care, commonly performed in PICUs. Among the patients in group A, only five required heart surgery within 30 days after the initial intervention. Avoiding PICU-care and open chest procedure can also lower the risk for nosocomial infections. CPB is associated with a known risk for acute kidney injury, which three patients in our material suffered from all of whom had gone through surgery and CPB [33, 34].

The approach regarding supplementary procedures during the initial intervention differed in the two cohorts. In group B an additional procedure, such as but not limited to, an extra source for pulmonary blood flow was added during the initial intervention based on preoperative morphologic traits while in group A an additional procedure was planned for in a second session or decided on when the patient was not able to be weaned of prostaglandin. There was a tendency towards fewer reinterventions within 30 days after the initial intervention among patients who underwent surgical intervention initially (*p*-value 0.34), consistent with previous studies [35, 36]. Regardless of the initial intervention technique, the most common reintervention aimed to augment pulmonary blood flow. Among patients initially undergoing heart surgery, nine out of 16 (56%) received an additional source of pulmonary blood flow during their first intervention. This treatment strategy may, at least partially, account for the lower rate of reinterventions in this group. The patients that were treated with catheter-based intervention did, however, have a smaller PV *z*-score preoperatively (see Table 2, *p*-value 0.01), which in previous studies has shown to be a risk factor for reintervention [29]. Half of the patients (5 patients) that underwent a catheter-based intervention initially and needed a reintervention within 30 days could be managed with a new catheter-based intervention, insertion of a PDA-stent, and thereby avoiding CPB in the neonatal period. Comparisons between mBTS to PDA-shunts for patients with duct-dependent pulmonary circulation have shown that patients with PDA-stents compared to patients with a mBTS had a lower risk of mortality, shorter hospital stay, and more reinterventions [37–40]. Thus, placing a PDA-stent instead of an AP-shunt, with interventional technique, might be an increasingly attractive option for patients initially treated with a transcatheter approach. It is worth noting that patients treated with heart surgery often undergo additional procedures, in addition to valvulotomy, during the initial intervention. Therefore, counting reinterventions may not provide a conclusive overview of treatment outcomes. Looking specifically at the need for extra pulmonary blood flow within 30 days can offer a more accurate assessment of the overall situation. This study cannot, due to the retrospective nature of the study design, draw any conclusions regarding if patients treated with surgery have an increased need for supplementary procedures or if the difference in additional interventions between the two groups is based on institutional preference.

We did not identify any preoperative echocardiographic variables that could predict the need for reintervention within 30 days after the initial intervention. However, the compliance of the right ventricle, which is challenging to evaluate through echocardiographic examination, can affect the need for additional pulmonary blood flow. Reduced compliance may result from working against high resistance.

There was one perforation of the pericardium periprocedural during catheter-based intervention (5.6%) which is a low periprocedural complication rate comparing to the studies included in the review [2, 6–15, 17–20, 25–27, 29]. Notably, we observed no mortality in the entire study population during long-term follow-up.

The cost of the intervention has been the focus of several studies [11, 13, 14, 25]. In Sweden, with its publicly financed welfare system, the cost of the intervention is not a determining factor when choosing the type of intervention.

### Systematic Review of Current Literature

There is currently no consensus on specific morphologic features that universally determine outcome. Different morphologic traits, such as a large TR (when not associated with tricuspid valve dysplasia), a well-developed right ventricle, and a large tricuspid valve annulus, are believed to contribute to the possibility of biventricular circulation and reduced need for reintervention.

The studies included in this review have different inclusion criteria, follow-up durations and varying definitions of reintervention. Therefore, meaningful comparisons can only be made in terms of short-term outcomes. Moreover, the age range of patients varies across studies, from newborns to 1.5 years old [12, 24]. The use of different methods to calculate *z*-scores sometimes makes it challenging to replicate results [41]. Notably, all the studies included in this review considered the included patients to have favorable anatomy, with the smallest tricuspid valve *z*-score ranging from − 5 to − 1. Several unaccounted factors, such as prenatal diagnostic prerequisites, birthing settings, access to advanced neonatal care and pediatric intensive care units (PICUs), and the prevalence of resistant bacteria, may also influence the outcomes and need to be considered. The often small study populations where individual patient outcomes heavily affect the overall results for the group might also have impacted the sometimes conflicting results observed in the reviewed studies.

Only four of the included studies evaluated initial heart surgery [19, 21, 28, 30]. It is possible that the variations in publication frequency could be influenced by centers utilizing different intervention techniques, with a greater inclination towards publishing. Given that surgery is already an established treatment option, this factor may also impact the likelihood of publication. Alternatively, it could be that there are fewer centers that perform heart surgery compared to percutaneous treatments. Surgery offers the advantage of adaptability during the procedure, allowing surgeons to address potential issues in a single session and potentially reduce the need for reinterventions [35]. It is, however, associated with a risk for right ventricle failure and low cardiac output syndrome [3, 42]. CPB in the neonatal period is correlated with several risks [4, 43].

Percutaneous intervention offers the advantage of avoiding heart surgery and CPB during the neonatal period. It also offers the possibility to place a PDA-stent if needed. However, studies have shown higher rates of reinterventions compared to surgery and hybrid interventions, indicating that CPB might not be entirely avoided [23, 35]. The most serious complication associated with catheter-based treatment is a perforation of the right ventricle or of the pulmonary artery. Various intervention techniques have been proposed and evaluated to minimize the risk for perforating the surrounding tissue [7, 9, 11, 13–15, 17, 18, 20].

The hybrid approach avoids CPB while still offering the opportunity to place a mBTS if needed and it decreases the risk of perforating the right ventricle or the pulmonary artery.

Previous studies have suggested that decompressing the right ventricle and establishing blood flow over the pulmonary valve may improve the chances of achieving biventricular circulation by promoting right ventricle growth [23, 44]. The acceptable oxygen level varies and influences the decision regarding the need for additional pulmonary blood flow. There has been a proposed correlation between both prostaglandin (PGE1) treatment and duct-dependent pulmonary circulation and necrotizing enterocolitis, this could speak in favor for shorter PGE1 treatment and earlier establishment of additional pulmonary blood flow via a mBTS or a PDA-shunt [8, 45, 46].

### Strengths and limitations

#### Retrospective Population-Based Multicenter Study

The major strength of the study is the inclusion of all patients with PA-IVS born in Sweden for 13 years and the pseudo randomization design of the study. The heart defect is, however, rare, so the number of patients remains small, which may have influenced the lack of statistical significance observed for several variables, especially when further subgrouping would be desirable. The small study population motivates the combination with the systematic literature review and despite the limited study population safety for the procedures could still be demonstrated with statistical significance. The angiograms and echocardiograms have been performed and interpreted by different personnel and with different equipment which could make the results harder to compare but it also limits the risk of bias. Our results also suffer from missing data and incomplete follow-up, which should be taken into consideration when interpreting the findings.

#### Systematic Review of Current Literature

To our knowledge, this is the most comprehensive overview and review of the subject.

## Summary and Conclusion

In conclusion, the findings of this retrospective population-based multicenter study demonstrate that both catheter-based intervention and heart surgery are safe procedures with a low risk of complications. Our results are at, or above, international standard compared to the included studies in the systematic literature review. It is desirable to avoid cardiopulmonary bypass (CPB) and minimize the length of stay in the pediatric intensive care unit (PICU).

The systematic literature review displays that given the heterogeneity of the heart defect and the variability in standards among cardiac centers worldwide, it remains challenging to establish a definitive gold standard treatment. However, a catheter-based approach followed by conversion to a hybrid intervention, if necessary, offers an appealing alternative. This approach is less invasive than heart surgery and provides the flexibility to incorporate a PDA-shunt during the same session or put in a mBTS without using CBP.

## Data Availability

No datasets were generated or analysed during the current study.
